# Genome editing in the nematode *Caenorhabditis briggsae* using the CRISPR/Cas9 system

**DOI:** 10.1093/biomethods/bpaa003

**Published:** 2020-02-10

**Authors:** Elizabeth Culp, Cory Richman, Devika Sharanya, Nikita Jhaveri, Wouter van den Berg, Bhagwati P Gupta

**Affiliations:** Department of Biology, McMaster University, 1280 Main Street West, Hamilton, ON L8S-4K1, Canada

**Keywords:** CRISPR/Cas9, genome editing, nematode, *Caenorhabditis briggsae*

## Abstract

The CRISPR/Cas system has recently emerged as a powerful tool to engineer the genome of an organism. The system is adopted from bacteria where it confers immunity against invading foreign DNA. This work reports the first successful use of the CRISPR/Cas system in *Caenorhabditis briggsae* (a cousin of the well-known nematode *C. elegans*), to generate mutations via non-homologous end joining. We recovered deletion alleles of several conserved genes by microinjecting plasmids that express Cas9 endonuclease and an engineered CRISPR RNA corresponding to the DNA sequence to be cleaved. Evidence for somatic mutations and off-target mutations are also reported. Our approach allows for the generation of loss-of-function mutations in *C. briggsae* genes thereby facilitating a comparative study of gene function.

## Introduction

Linking genotype and phenotype is an important step in the characterization of a gene. Targeted genome editing, defined as the creation of alterations at specific sites in an organism’s genome, is a powerful means to study the relationship between gene and phenotype. Genome editing techniques are based on guiding an endonuclease to a specific target in the genome in order to generate a double strand break (DSB) [1–3]. Breaks are subsequently repaired by either error prone non-homologous end joining (NHEJ) or template-directed homologous recombination (HR) [4]. While the former introduces random mutations at the point of cleavage, the latter can be used to generate specific alterations based on the presence of a donor sequence. Although several technologies currently exist for genome editing, such as zinc finger nucleases (ZFN) and transcription activator-like effector nucleases (TALEN), these techniques leave room for improvement in their ease of use, as each new sequence to be targeted requires the labor-intensive process of generating a new protein construct [2].

Clustered, regularly interspaced, short palindromic repeats (CRISPR) and CRISPR-associated (Cas) systems are known to have evolved in archaea and bacteria to serve as adaptive immune mechanisms to defend against foreign plasmids and viral DNA [5, 6]. Although, a recent study reported the existence of an inverse strategy in *Vibrio cholerae* for RNA-guided integration of Tn7-like transposons into the host genome [7]. The power of CRISPR/Cas to edit genomic DNA in a targeted manner has led to the development of protocols to alter genes in eukaryotes. First, a 20 bp sequence in a gene of interest is selected to act as a template to produce a guide for the *Streptococcus* *pyogenes* nuclease, Cas9. This sequence, termed the CRISPR RNA (crRNA), has only one requirement, namely that it must precede a Protospacer Adjacent Motif (PAM) of the form 3′NGG [8] when interacting with the Cas9 protein, although recent engineered versions of the Cas9 protein can bind to alternative PAM sequences [9]. Next, a second RNA molecule termed the trans-activating crRNA (tracrRNA), is used for binding to Cas9 [8]. In one CRISPR/Cas9 strategy, crRNA and tracrRNA sequences are fused into a single guide RNA (sgRNA) [10]. By expressing this sgRNA along with Cas9 in germ line cells, heritable genome mutations can be created.

In the case of nematodes, CRISPR/Cas9 system was initially established in *C. elegans* and *Pristionchus pacificus* [2, 11, 12]. Subsequently, the technique was also shown to work in *C. remanei* [13] and parasitic nematodes such as *Strongyloides spp.* and *Auanema freiburgensis* [14–17]. CRISPR mutants in *C. elegans* were generated initially by injecting plasmids encoding genes for Cas9 and a pre-fused sgRNA into the gonad of adult hermaphrodites [12]. A modified Cas9 was used that included a SV40 NLS to ensure nuclear localization and expression under an *eft-3* translation elongation factor promoter, chosen for its effectiveness in germ line expression. A detailed protocol has also been published [18].

Adaptation of CRISPR/Cas9 to *Caenorhabditis briggsae*, a species that is closely related to *C. elegans,* would provide a powerful tool to investigate the function of any given gene. *C. briggsae* is used routinely by many laboratories in comparative evolutionary studies. The two animals diverged over 25 million years ago yet share similar morphology [19]. A comparison of the genomes has revealed that roughly one-quarter of their genes lack clear orthologs including many that are highly divergent and species-specific [20]. This suggests that underlying gene networks have evolved substantially without an obvious change in phenotype [21]. Such changes are likely to have significant impacts and may confer unique advantages on animals to withstand genetic and environmental fluctuations. By generating mutations in *C. briggsae* genes and characterizing phenotypes, we can learn the functional relevance of genomic differences, including any alterations in genetic pathways and developmental mechanisms between the two species. With this goal in mind, we set out to develop a method for using this system in *C. briggsae*.

## Materials and methods

The wild type AF16 strain was used as a reference strain in all experiments. Strains generated as part of this study include DY503 *Cbr-unc-22(bh29)*, DY504 *Cbr-dpy-1(bh30),* DY530 *Cbr-bar-1(bh31),* DY544 *Cbr-unc-119(bh34)*, and DY545 *Cbr-unc-119(bh35).*

The plasmids containing the *C. elegans* U6 promoter and sgRNA target sequences were generated by site-directed mutagenesis. This was accomplished using either two-step overlap-extension PCR on a *pU6::Cbr-unc-119_sgRNA* template (gift from John Calarco, Addgene plasmid #46169) [12], or Q5 site-directed mutagenesis on a *pU6::Cbr-lin-10_sgRNA* template [22] using the NEB Q5 site-directed mutagenesis kit (E0554). The target site substitution was confirmed by *AclI* digestion. See Supplementary Tables S1 and S2 for sgRNA sites and primers used in this study. The sgRNAs were expressed under a U6 small nuclear RNA polymerase III promoter, chosen for its ability to drive expression of small RNAs. As the optimal expression from this promoter requires the first base to be a purine, the sgRNA target sequence is restricted to the form (G/A)(N)_19_NGG [12, 23].

The plasmids containing sgRNA and Cas9 (*Peft-3::Cas9-SV40 NLS::tbb-2 3′UTR*, also from John Calarco, Addgene #46168) were injected into the germline of young adults using standard methods established for generating transgenic animals [24]. A plasmid carrying *GFP*, expressed in the pharynx (*myo-2::GFP*), was included as a co-injection marker. F1 progeny displaying fluorescence in pharynx were isolated onto separate plates and allowed to propagate. Injection mixes contained *pU6::sgRNA* (100 ng/µl), *Peft-3::Cas9-SV40 NLS::tbb-2 3′UTR* (100 ng/µl), and *myo-2::GFP* (10 ng/µl). For the PCR-based assay [22] F1s were allowed to lay eggs for 24–36 hours, and then picked and lysed in pools of two. A region of the genomic DNA spanning the sgRNA site (∼200 bp) was amplified and examined on a 4% high-resolution agarose gel (Invitrogen UltraPure Agarose-1000, catalog #16550-100) for changes in band sizes (Supplementary Figure S2).

For HR method-based gene editing experiments involving reporter genes, two different attempts were made. In one case, the donor vector *myo-2::dsRED::unc-54 3′UTR* was designed to insert a *myo-2::dsRED* reporter into the *Cbr-bar-1* (Supplementary Figure S1A) [25]*.* The vector contained a 2 kb transgene flanked on either side by 1 kb of sequence homologous to *Cbr-bar-1* (Gibson Assembly Cloning Kit NEB catalog #E5510). The templates were included in the injection mix (donor plasmid 200 ng/µl, linear PCR amplicons 50 ng/µl, single-stranded oligonucleotides 30 ng/µl) along with other DNA components as mentioned above. The second set of attempts involved the use of *GFP* and *dsRED* fragments. The double-stranded linear donor templates of *GFP* (864 bp) and *dsRED* (830 bp) containing short microhomology arms were generated by PCR to create translational fusions with *Cbr-bar-1 (GFP)* (Supplementary Figure S1C)*, Cbr-lin-15B (GFP)*, and *Cbr-vit-2 (dsRED)* (similar to *Cbr-bar-1*, figure not shown).

## Results

We used CRISPR/Cas9 in *C. briggsae* initially to generate targeted loss-of-function mutations by employing NHEJ. For this, two conserved genes were chosen based on visible phenotypes, *Cbr-dpy-1,* a cuticle protein whose lack of function causes dumpy (Dpy) phenotype, and *Cbr-unc-22,* a twitchin homolog that exhibits uncoordinated (Unc) phenotype when mutated [26–28]. Target sgRNA sequences following the form G/A(N)_19_NGG were searched for in the exonic regions of these genes using the ZiFiT Targeter Version 4.2 software [29]. The sgRNA sites were screened based on predicted efficiency using empirically based scoring algorithms. Off-target sites were minimized using the sgRNAcas9 software package developed by Xie *et al.* [30].

Following microinjection of CRISPR/Cas9 plasmid mix, F2 worms were screened for desired phenotypes. We successfully isolated mutants for both *Cbr-dpy-1* and *Cbr-unc-22* at comparable frequencies to those observed in *C. elegans* (Table 1) [12]. All mutations recovered were heritable. Sequencing of the alleles of each of these genes revealed insertions and deletions at the sgRNA target sites (Table 2). The phenotypes of mutant animals are indistinguishable from those in *C. elegans* corresponding to orthologous genes, demonstrating conservation of gene function. Together, these results show that the CRISPR/Cas9 system works in *C. briggsae* and can utilize conserved *C. elegans* promoters to express sgRNAs and Cas9.

**Table 1:** Phenotypes of transgenic animals generated using the CRISPR/Cas9 technique

Screening approach	Targeted gene	3′ Target bases	Visible phenotype	Frequency of mutations	Animals screened
Phenotypic screening	*Cbr-bar-1*	GG	Egl	9.5%	22
	*Cbr-dpy-1*	GA	Dpy	2.8%	35
	*Cbr-lin-2*	UA	Vul	0	40
	*Cbr-lin-7*	GA	Vul^a^	0	44
	*Cbr-lin-10*	AC	Vul	0	161
	*Cbr-lin-17*	AC	Bivulva	0	63
	*Cbr-lin-17* (linear sgRNA)	AC	Bivulva^b^	0	3
	*Cbr-lin-18*	AG	Bivulva^c^	0	65
	*Cbr-unc-22*	UC	Unc	2.5%	40
	*Cbr-unc-119* (sgRNA #1)	TT	Unc	0	48
	*Cbr-unc-119* (sgRNA #2)	GG	Unc	11.1%	54
PCR-based screening	*Cbr-lin-7*	GA	Vul	0	56
	*Cbr-lin-10*	AC	Vul	0	126
	*Cbr-vit-2*	AG	WT^d^	1.3%	78

The 3′ target bases are those at positions 19 and 20 in the sgRNA target sequence. ^a^One F2 showed Dpy phenotype. ^b^3 bivulva worms were recovered in F3 but the phenotype was not heritable. ^c^One F2 showed protruding vulva (Pvl) phenotype. ^d^Wild type, based on the *C. elegans vit-2* mutant phenotype.

**Table 2.** Alleles generated by the CRISPR/Cas9 approach

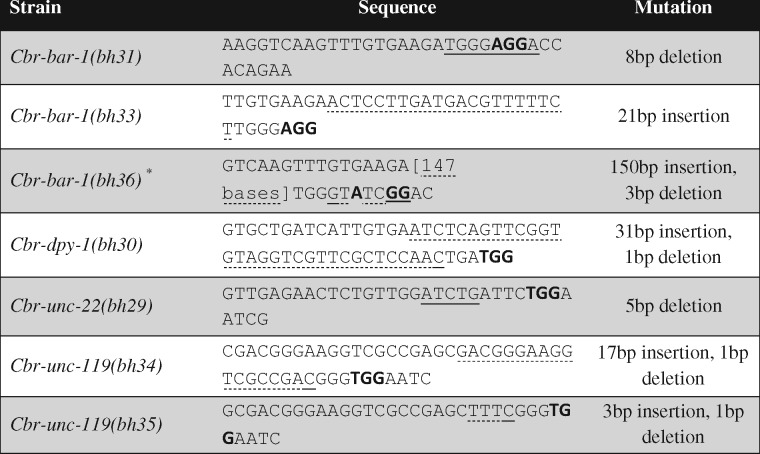


The DNA sequence includes the sgRNA target. The PAM site is bolded. Insertion and deletion sequences are underlined (dotted underline: insertion, solid underline: deletion). For clarity, the 147 base pair inserted sequence in *bh36* allele has been omitted. This long sequence matches with the *E. coli* gene EF-Tu. ^a^The allele was recovered in a separate screen along with another allele *bh32* that has a small deletion. The exact base change in *bh32* has not been determined.

Next, six other conserved genes of “*lin*” and “*vit*” classes were targeted (*Cbr-lin-2, Cbr-lin-7, Cbr-lin-10, Cbr-lin-17, Cbr-lin-18*, and *Cbr-vit-2*). Orthologs of all these genes, except *Cbr-vit-2* (member of the *vitellogenin* family), encode Wnt and Ras pathway components (www.wormbase.org). In some cases, mutations were recovered as determined by phenotype and PCR-based screening, but none were found to be heritable (Table 1). It is unclear whether it was due to sgRNAs being nonfunctional, less efficient or requiring much larger F1s to be screened. Similar results were previously reported in *C. elegans* [31]. In one case, *Cbr-lin-17*, we sequenced the animal that showed bi-vulva phenotype and found possible evidence for a somatic mutation (T/A transversion causing M482L substitution). The bi-vulva phenotype in this line was lost in subsequent generations. Evidence of somatic mutations has also been described in *C. elegans* [31].

Interestingly, our screens also recovered worms with unexpected phenotypes, e.g., dumpy in a *Cbr-lin-7* screen (Table 1). Sequencing of these worms revealed no disruption in targeted genes, raising the possibility of off-target effects of CRISPR/Cas9.

The sgRNAs with a 3′GG motif at positions 19 and 20 are shown to significantly enhance the efficiency of targeted mutations in *C. elegans* [31]. To test whether a similar sequence structure could be effective in *C. briggsae* we selected two conserved genes *Cbr-unc-119* and *Cbr-bar-1*. We successfully isolated heritable mutations in both these genes that exhibit defects. Mutations in *Cbr-unc-119* with Unc phenotype were recovered at a frequency of 11.1% (Tables 1 and 2). In contrast, another sgRNA for *Cbr-unc-119* that lacked 3′ GG motif did not give rise to any mutant line (Table 1). Since the animals were not screened by PCR, the evidence for somatic mutations is lacking. In the case of *Cbr-bar-1,* a β-catenin homolog [32], the 3′GG motif sgRNA resulted in a disruption efficiency of 9.5% (Tables 1 and 2). The enhanced efficiency of the 3′GG motif sgRNA sites for these two genes suggests that such an approach in *C. briggsae* could improve the frequency of targeted mutations in genes of interest.

In addition to the CRISPR-mediated NHEJ approach, we also attempted the HR method of gene editing in *C. briggsae.* In *C. elegans*, HR CRISPR has been successfully demonstrated that utilizes donor templates having short homology arms [9, 22, 33]. In our case, donor templates were designed to either disrupt a gene (by inserting a single-stranded oligonucleotide) or tag genes using double-stranded linear PCR amplicons (or plasmids) of fluorescent reporters (*GFP* and *dsRED*). Specifically, the single strand oligonucleotide donor templates were intended to insert a 22-bp sequence containing an *NcoI* restriction enzyme site into *Cbr-bar-1* and a Synmuv class B ortholog *Cbr-lin-15B* (www.wormbase.org) (Supplementary Figure S1B). Homology arms of length 75 and 49 bases were chosen directly overlapping the sgRNA site, based on previous results [22]. The fluorescent reporters were tested in the case of *Cbr-bar-1, Cbr-lin-15B* and *Cbr-vit-2* using long as well as short homology arms (see *Cbr-bar-1* as one example in Supplementary Figures S1A, C). Although none of these HR approaches were successful, in some cases we did observe expected genomic changes in F1 and F2 animals as determined by sequencing, which were not inherited in subsequent generations (Table 3; Supplementary Figure S2).

**Table 3.** Genome editing events detected using CRISPR-mediated HR

Targeted gene	Expected phenotype	sgRNA efficiency	HR efficiency
*Cbr-bar-1*	Egl	25/219 (11.4%)	0/219
*Cbr-bar-1*	Egl	18/211 (8.5%)	0/211
*Cbr-bar-1*	Egl	Not Determined	0/202
*Cbr-lin-15B*	WT^a^	Not Determined	0/68^b^
*Cbr-vit-2*	WT^a^	1/75 (1.3%)	0/75

The sgRNA efficiency shows all genome editing events, including those repaired by NHEJ and HR, based on phenotypic and PCR-based screens. HR efficiency indicates the number of HR events detected in F2 out of the total F1s screened.

^a^Wild type, based on the phenotype of *C. elegans* orthologs.

^b^A total of 79 F1s were recovered, of which 68 were tested by PCR.

## Discussion

In this article, we have demonstrated that the CRISPR/Cas9 system can be effectively employed in *C. briggsae* to alter a gene of interest. Similar to *C. elegans* the 3′ GG motif appears to increase the frequency of NHEJ events. Interestingly, we observed a significant bias toward insertion NHEJ events in *C. briggsae.* Of the total of 8 alleles recovered, for 4 different genes, 62% had insertion of bases of varying length (range 3–150). Similar screens in *C. elegans* have reported 26% frequency of such events (n = 86 from five different studies) [12, 31, 34–36]. More work is needed to ascertain if such a bias in *C. briggsae* holds true in a larger sample size.

In the case of “*lin*” and “*vit*” class of genes, use of CRISPR was effective in certain cases where somatic mutations were recovered. However, no germline mutant animals were found. Moreover, neither the HR-based CRISPR approach nor PCR-based screening yielded success. There might be several reasons for this. One, we used heterologous promoters from *C. elegans* to express Cas9 and sgRNA in *C. briggsae*. This might have affected the levels of nucleases and guide RNAs needed for CRISPR genome editing process. In the future, endogenous promoters should be tested, similar to an earlier work involving TALEN-based genome editing in *C. briggsae* [37]. Alternatively, purified Cas9 protein and sgRNAs could be injected instead of plasmids as demonstrated by a recent study [38]. Two, it may be that certain genes are difficult to mutate. We had good success with genes encoding cytoplasmic and structural proteins (*Cbr-dpy-1, Cbr-unc-22, Cbr-bar-1*, and *Cbr-unc-119*) but not with “*lin*” and “*vit*” family members. Few other studies have also reported generation of CRISPR alleles of *C. briggsae* genes that code for cytoplasmic proteins namely *Cbr-met-2* [39], *Cbr-fem-3* [40], and *Cbr-ben-1* [41]. In the future, a wider range of gene families should be targeted by CRISPR method to examine any biases. Three, it may be that a larger number of *C. briggsae* animals need to be injected to obtain mutations. This would be consistent with observations in our lab that generating transgenic lines in *C. briggsae* is less efficient compared to *C. elegans* (BG, unpublished data). Additional possibilities are also likely.

In the case of HR CRISPR, a failure to obtain transgenic animals may also be due to the large size of *GFP* and *dsRED* donor templates. Paix *et al.* [22] had earlier reported that a large insert can be introduced into the genome with the CRISPR method, however with very low success rate (typically 1% or less, highest 4%).

In recent years, several modified versions of the CRISPR technique have been reported in *C. elegans* that may be tested in the *C. briggsae* system to increase the efficiency of gene editing. The resources include a standardized toolkit for plasmid production [42] and guide RNA delivery through guide RNA plasmid-carrying bacteria [43]. In terms of applications to make edits into genomic DNA, studies have reported the use of co-conversion markers to enable rapid screening of candidate lines [41, 44], generation of mutations by inserting a universally applicable cassette of STOP-codons [45], and insertion of large genomic fragments as well as reporters (e.g., GFP) through a two-step, co-CRISPR pipeline [38]. These developments hold promise for more effective and wider applications of CRISPR/Cas9 in *C. briggsae* and other nematodes.

Also, several cases of off-target mutations were detected in our screens. Off-target effects of CRISPR/Cas gene editing have been observed in *C. elegans* as well as several other models including *Drosophila,* mice, zebrafish, and human cell lines [46–49]. High concentrations of either the guide RNA: Cas9 complexes or the Cas9 enzyme could be the cause of high off-target mutation frequency [50].

The CRISPR/Cas9 procedure described here provides a useful means to investigate the functions of conserved as well as divergent genes in *C. briggsae*. This promises to accelerate comparative studies with *C. elegans* thereby leading to a greater understanding of the flexibility of genetic and molecular mechanisms during animal development.

## Supplementary Material

bpaa003_Supplementary_Data
