# Supplementary material for: Genome editing in the nematode Caenorhabditis briggsae using the CRISPR/Cas9 system
Source: Biol Methods Protoc. 2020 Feb 10;5(1):bpaa003. doi: 10.1093/biomethods/bpaa003 (PMC7200835; doi:10.1093/biomethods/bpaa003)

**Supplementary Materials**

**Table S1. sgRNA target sites.**

| **Gene** | **sgRNA Target** |
| --- | --- |
| *Cbr-bar-1* | GTCAAGTTTGTGAAGATGGG**AGG** |
| *Cbr-dpy-1* | GTGCTGATCATTGTGACTGA**TGG** |
| *Cbr-lin-2* | GATTAGAGACAAAGAGCATA**TGG** |
| *Cbr-lin-7* | GGTTCGAGAGGTTTATGAGA**CGG** |
| *Cbr-lin-10* | GTCCCACAGCAACAAGAAAC**AGG** |
| *Cbr-lin-15B* | GCCGTCAACAACTACACCTA**TGG** |
| *Cbr-lin-17* | GTGTTGTCCAGTTTGACCAC**TGG** |
| *Cbr-lin-18* | GCTCCGGAAGCAATTGCTAG**AGG** |
| *Cbr-unc-22* | AACTCTGTTGGATCTGATTC**TGG** |
| *Cbr-unc-119* #1 | GGAAGTGCTAAAACGTCGTT**CGG** |
| *Cbr-unc-119* #2 | GGGAAGGTCGCCGAGCCGGG**TGG** |
| *Cbr-vit-2* | AATGATGCACACCCGCCCAG**AGG** |
| Bold indicates the PAM site. | |

**Table S2. List of Primers.**

| **Gene** | **Purpose** | **Name** | **Direction** | **Sequence** |
| --- | --- | --- | --- | --- |
| *Cbr-bar-1* | sgRNA (OE) | GL964 | F | GTCAAGTTTGTGAAGATGGGGTTTTAGAGCTAGAAATAGCAAGTTA |
|  |  | GL965 | R | CCCATCTTCACAAACTTGACAAACATTTAGATTTGCAATTCAATTATATAG |
|  | 5’ homology arm for Gibson Assembly | GL991 | F | CGAGGTCGACGGTATCGATATCTGAGCAGCCACGCTAA |
|  |  | GL992 | R | TCTCTACTTGTCTAGAAGCTAAGATTATGCGGTAAATAGTCTAATAATTG |
|  | 3’ homology arm for Gibson Assembly | GL1001 | F | AAGTCGAAAAAAATTAAGCTTTTTGAAAGACACTATATTTGGCTCG |
|  |  | GL1002 | R | GCTGCAGGAATTCGATATCAACCTAGTTATCAACCATGACGATAC |
|  | Sequencing | GL1009 | F | CATCTTGCTAGGCACATCACTTATA |
|  |  | GL1010 | R | GGCAACAAGATGCGATCATTG |
|  | PCR amplicon for direct HR | GL1039 | F | GAATACTGACCAAAAGGTCAAGTTTGTGAAGATGGGAAAAGGAGAAGAACTTTTCACTGG |
|  |  | GL1040 | R | GTTGTTGCAGAATATGGAGCAGTTTCTGTGGTCCCTATTTGTATAGTTCATCCATGCC |
|  | single stranded oligonucleotide donor template | GL1058 | N/A | CTTCTTCCTGTTATCGTCGACTTGATCAGAGTTCTATGTGAAAAGAATACTGACCAAAAGGTCAAGTTTGTGAAGGACCATGGGCTGGGAGGGTAAGATGGGAGGACCACAGAAACTGCTCCATATTCTGCAACAACGAGGATATG |
|  | PCR screening | GL1059 | R | CATGGGCTGGGAGGGTAAG |
| *Cbr-dpy-1* | PCR stitching | GL954 | F | GTGCTGATCATTGTGACTGAGTTTTAGAGCTAGAAATAGCAAGTTA |
|  |  | GL955 | R | TCAGTCACAATGATCAGCACAAACATTTAGATTTGCAATTCAATTATATAG |
|  | Sequencing | GL968 | F | GGAGGAAGCCAACTCACCAAG |
|  |  | GL969 | R | CAGCTCGATTTCCAGACAATTC |
| *Cbr-lin-2* | sgRNA (OE) | GL974 | F | GATTAGAGACAAAGAGCATAGTTTTAGAGCTAGAAATAGCAAGTT |
|  |  | GL975 | R | TATGCTCTTTGTCTCTAATCAAACATTTAGATTTGCAATTCAATTATATAG |
| *Cbr-lin-7* | sgRNA (OE) | GL976 | F | GGTTCGAGAGGTTTATGAGAGTTTTAGAGCTAGAAATAGCAAGTT |
|  |  | GL977 | R | TCTCATAAACCTCTCGAACCAAACATTTAGATTTGCAATTCAATTATATAG |
|  | PCR-based screening and sequencing | GL1024 | F | TGGGCCAATTCTATATCGATT |
|  |  | GL1025 | R | TTGCAGTCGAAATATGGGAT |
| *Cbr-lin-10* | sgRNA (OE) | GL978 | F | GTCCCACAGCAACAAGAAACGTTTTAGAGCTAGAAATAGCAAGTT |
|  |  | GL979 | R | GTTTCTTGTTGCTGTGGGACAAACATTTAGATTTGCAATTCAATTATATAG |
|  | PCR-based screening | GL1043 | F | CAAGCCAATGCATAATATGCTCAATAG |
|  |  | GL1044 | R | CTTCTTGATATTGTGCCGGCGAG |
| *Cbr-lin-15B* | sgRNA #1 | GL1065 | F | GCCGTCAACAACTACACCTAGTTTTAGAGCTAGAAATAGCAAG |
|  | sgRNA #2 | GL1066 | F | GTTGTTGACGGCACGACGGAGTTTTAGAGCTAGAAATAGCAAG |
|  | PCR-based screening | GL1067 | F | CGACGATCAGAAGTACCTCGTG |
|  |  | GL1068 | R | CGGCATCCTGTCGAATGTATTTC |
|  | Single stranded oligonucleotide donor template | GL1064 | N/A | GTATCGAAGCGGAAACATTGCTCACTTCCATGTGTCGTGCCCATGGGCTGGGAGGGTAAGCTAGTCAACAACTACACCTATCGAACTGTGAAATTCAGTAACATCGTCTGCCCCAATGAATCG |
| *Cbr-lin-17* | sgRNA #1 (OE) | GL960 | F | GTGTTGTCCAGTTTGACCACGTTTTAGAGCTAGAAATAGCAAGTTA |
|  |  | GL961 | R | GTGGTCAAACTGGACAACACAAACATTTAGATTTGCAATTCAATTATATAG |
|  | sgRNA #2 | GL1076 | F | GGAACTTGCTTTATTGTCGGGTTTTAGAGCTAGAAATAGCAAG |
|  | PCR-based screening and sequencing | GL1077 | F | CGGTGGGAAACCTGAATTCGATC |
|  |  | GL1078 | R | GTATAGTCTCACCCTTGTTCTG |
| *Cbr-lin-18* | sgRNA (OE) | GL962 | F | GCTCCGGAAGCAATTGCTAGGTTTTAGAGCTAGAAATAGCAAGTTA |
|  |  | GL963 | R | CTAGCAATTGCTTCCGGAGCAAACATTTAGATTTGCAATTCAATTATATAG |
|  | Sequencing | GL1011 | F | GCTCTTGCCACTCAGTTATCC |
|  |  | GL1012 | R | CACATGAGCACTCCTAGGGAC |
| *Cbr-unc-22* | PCR stitching | GL942 | F | AACTCTGTTGGATCTGATTCGTTTTAGAGCTAGAAATAGCAAGTTA |
|  |  | GL943 | R | GAATCAGATCCAACAGAGTTAAACATTTAGATTTGCAATTCAATTATATAG |
|  | Sequencing | GL966 | F | GGAGAAACCGTTGAGTTGAAG |
|  |  | GL967 | R | CCATGATCCTCCCATAGCTTC |
| *Cbr-unc-119* | sgRNA #1 | GL1047 | F | GGAAGTGCTAAAACGTCGTTGTTTTAGAGCTAGAAATAGCAAG |
|  | sgRNA #2 | GL1079 | F | GGGAAGGTCGCCGAGCCGGGGTTTTAGAGCTAGAAATAGCAAG |
|  | Sequencing | GL1099 | F | GGCACCCTCTAATTACCATT |
|  |  | GL1100 | R | GATTCCTTGTTCGGTGCTTG |
|  | sgRNA (OE) | GL940 | F | CGGGAATTCCTCCAAGAACTCGTACAAAAATGCTCT |
|  |  | GL941 | R | CGGAAGCTTCACAGCCGACTATGTTTGGCGT |
|  | sgRNA (Q5) | GL1048 | R | AAACATTTAGATTTGCAATTCAATTATAT |
| *Cbr-vit-2* | sgRNA #1 (OE) | GL1029 | F | AATGATGCACACCCGCCCAGGTTTTAGAGCTAGAAATAGCAAGTT |
|  |  | GL1030 | R | CTGGGCGGGTGTGCATCATTAAACATTTAGATTTGCAATTCAATTATATAG |
|  | sgRNA #2 (OE) | GL1033 | F | GGCGGGCCTCGACGGTCAAAGTTTTAGAGCTAGAAATAGCAAGTT |
|  |  | GL1034 | R | TTTGACCGTCGAGGCCCGCCAAACATTTAGATTTGCAATTCAATTATATAG |
|  | PCR-based screening (sgRNA #1) | GL1049 | F | ACCGTCAATACGAGCCAGAA |
|  |  | GL1050 | R | TAGCACACTCAGTGGCAACA |
|  | PCR amplicon to direct HR | GL1053 | F | GCCAGAAATCCGCATTCTTGCTCTCTGGAGAATGATGCACATGGTGCGCTCCTCCAAGAA |
|  |  | GL1054 | R | GAGAGACGACTTGAACGAGGAGTGGCTCCTCTGGGCGGGTGAACTCAGTTTAAACTTACT |
| F indicates forward, R indicates reverse. sgRNA plasmids were either generated by overlap extension PCR (OE), or Q5 site directed mutagenesis (Q5). | | | | |

**Figure S1. Donor sequence approaches generated as templates for HR for Cbr-bar-1.**

Templates may take the form of a donor vector (A), ssODN (B) or PCR amplicons (C). Blue letters represent the sgRNA target sequence while red letters represent the PAM site.


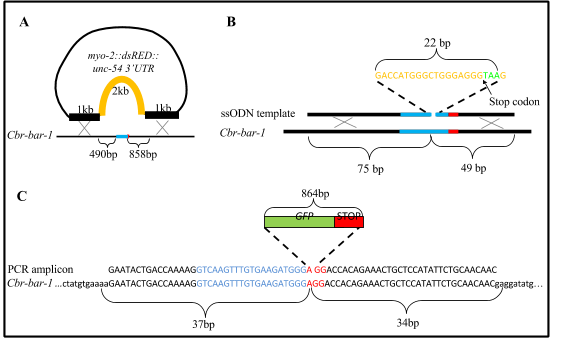


**Figure S2. PCR amplicons of the *Cbr-vit-2* genomic region flanking the sgRNA target site*.***

An insertion can be seen near the 204 bp fragment (sgRNA site #1), marked with *, in the first lane. The inset shows DNA bands in the same lane, captured after a longer period of electrophoresis. The insertion is faint but visible (white arrow and star).


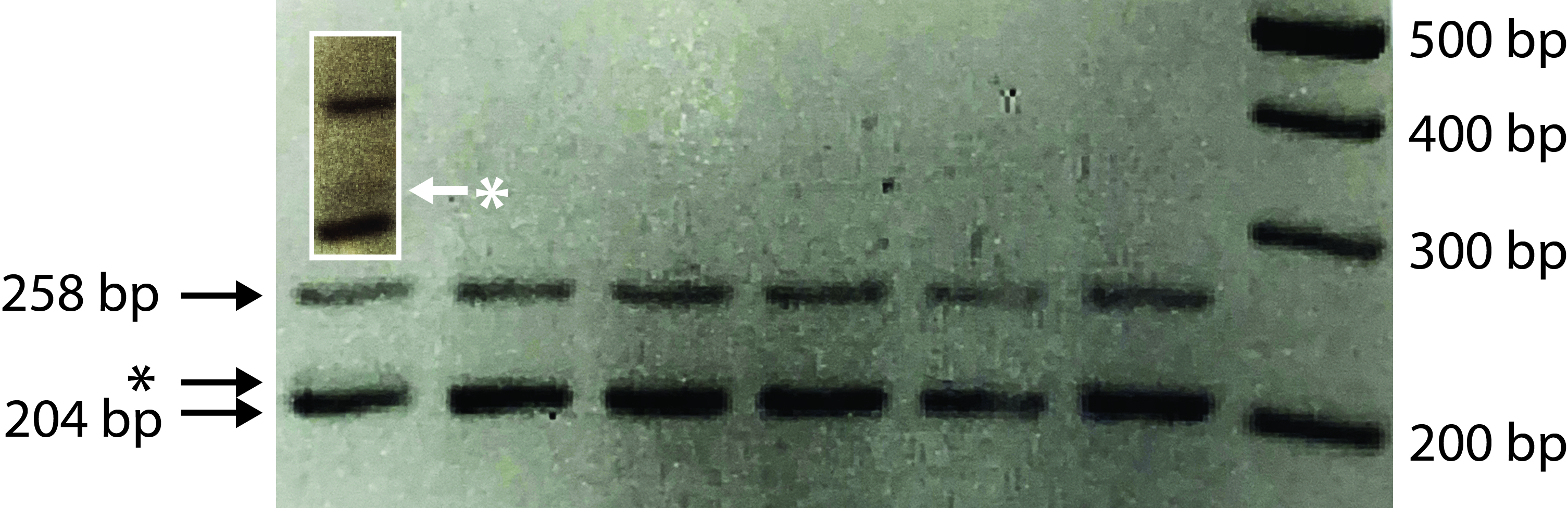

Supplement: bpaa003_Supplementary_Data [file bpaa003_supplementary_data.docx]
